# Group B Streptococcus Meningitis Following Elective Termination of Pregnancy: Two Case Reports

**DOI:** 10.1155/S1064744995000172

**Published:** 1995

**Authors:** Mark A. Walker, S. Gene McNeeley

**Affiliations:** Department of Obstetrics and Gynecology Hutzel Hospital Wayne State University School of Medicine 4707 St. Antoine Detroit MI 48201 USA

## Abstract

*Background:* Although maternal group B streptococcus (GBS) infections are common, serious infections are rare with prompt diagnosis and treatment. We present 2 cases of GBS meningitis occurring 3 and 10 days after elective abortion. In the first patient, GBS meningitis was definitely related to the elective termination. In the second patient, however, no evidence for a causal relationship could be established and can only be presumed.

*Case:* The patients presented to the emergency department with headache, altered mental status, and fever. Their physical examinations were consistent with meningitis and confirmed by cerebrospinal fluid (CSF) analysis. One patient recovered completely and the other developed severe bilateral hearing loss.

*Conclusion:* GBS meningitis is rare, occurring in men and women. When associated with pregnancy, most cases present within 48–72 h of delivery or abortion.


Infectious Diseases in Obstetrics and Gynecology 2:275–278 (1995)
© 1995 Wiley-Liss, Inc.

### Group B Streptococcus Meningitis Following Elective Termination of Pregnancy: Two Case Reports

**Mark A. Walker and S. Gene McNeeley**

*Department of Obstetrics and Gynecology, Hutzel Hospital, Wayne State University School of Medicine, Detroit, MI*

Abstract

***Background:*** **Although maternal group B streptococcus (GBS) infections are common, serious infections are rare with prompt diagnosis and treatment. We present 2 cases of GBS meningitis occurring 3 and 10 days after elective abortion. In the first patient, GBS meningitis was definitely related to the elective termination. In the second patient, however, no evidence for a causal relationship could be established and can only be presumed.**

***Case:*** **The patients presented to the emergency department with headache, altered mental status, and fever. Their physical examinations were consistent with meningitis and confirmed by cerebrospinal fluid (CSF) analysis. One patient recovered completely and the other developed severe bilateral hearing loss.**

***Conclusion:*** **GBS meningitis is rare, occurring in men and women. When associated with pregnancy, most cases present within 48–72 h of delivery or abortion.** © 1995 Wiley-Liss, Inc.

Key words

*Streptococcus agalactiae*, meningitis, abortion

Approximately 1.5 million abortions are performed annually in the United States, making termination of pregnancy one of the most commonly performed procedures. Infection is one of the most common complications of abortion, occurring in 0.1–30% of women.[1] Genital colonization with *Neisseria gonorrhoeae* or *Chlamydia trachomatis* or the presence of bacterial vaginosis predisposes a patient to postabortion endometritis. Antibiotics have been shown effective in reducing the incidence of endometritis, especially in patients who have chlamydia or bacterial vaginosis.[2,3] Group B streptococcus (GBS) colonization of the nonpregnant woman is infrequently associated with infections.[4] The association of maternal GBS colonization with neonatal morbidity and mortality is well documented.[5,6] Although GBS is a major cause of maternal morbidity, serious infections such as endocarditis and meningitis are rare if maternal infections are promptly diagnosed and treated. We describe 2 cases of maternal GBS meningitis following abortion. In the first patient, GBS meningitis was definitely related to the elective termination. In the second patient, however, no evidence for a causal relationship could be established and can only be presumed.

### CASE 1

A 36-year-old $G_2$ $P_1$ at 20 weeks gestation underwent an uneventful elective dilatation and evacuation. Other than a term vaginal delivery, the patient's medical history was unremarkable. She was

Address correspondence/reprint requests to Dr. S. Gene McNeeley, Department of Obstetrics and Gynecology, Hutzel Hospital, 4707 St. Antoine, Detroit, MI 48201.

Received October 4, 1994
Accepted January 5, 1995

discharged home on methergine, 0.2 mg 3 times daily; ibuprofen, 400 mg 4–6 h as needed; and tetracycline, 500 mg 4 times daily for 5 days, which she stated she took accordingly. On the second postoperative day, she began experiencing a generalized headache and dizziness. An acquaintance noticed that she was walking into the walls and had fallen once. The gynecologist who performed the abortion was informed of her problems. He instructed her to stop the methergine and, if the symptoms persisted or worsened, to go to the emergency room. With a worsening headache and altered mental status, she was taken to the emergency room the following day by a family member.

The patient was unable to respond to questions from the emergency personnel. She was responsive only to strong stimuli. On examination, her vital signs were stable and her oral temperature was 38.2°C. The head examination was unremarkable. Nuchal rigidity was present. An examination of the heart, lungs, and abdomen was within normal limits. A pelvic examination revealed a 10-week-size uterus with a purulent discharge and a closed os. A neurological examination showed hyperreflexia, downward plantar reflex, and a positive Kernig's sign.

Computerized tomography of the head showed no evidence of bleeding or edema. Her serum hemoglobin was 8.0 g/dl and WBC count was 19,200/mm$^3$. A lumbar puncture was performed and purulent fluid noted. The results of the cerebrospinal fluid (CSF) included WBC count 3,762/mm$^3$ with 94% neutrophils; glucose $<$ 20 g/dl; protein 213 g/dl; nonreactive VDRL; and negative antigen testing for 10 common organisms except for a positive test for GBS (Directigen, Bectin Dickinson, Baltimore, MD). The CSF culture showed no growth. The serum quantitative beta-HCG was 96 mIU. Blood and cervical cultures grew GBS. A cervical swab for *C. trachomatis* was negative.

The patient received 1 dose of IV cefotaxime before the GBS antigen test was reported positive, after which she was changed to aqueous penicillin G, 2 million units IV q 2 h for 14 days. On hospital day 3, a suction dilatation and curettage was performed for ultrasound findings consistent with retained products of conception which was confirmed by histology. She recovered completely with the exception of severe bilateral hearing loss.

### CASE 2

A 33-year-old $G_5$ $P_1$ $Ab_4$ underwent an elective termination at approximately 7 weeks gestation at her physician's office. She was given prescriptions for methergine, 0.2 mg 3 times daily; ibuprofen, 400 mg q 4–6 h as needed; and tetracycline, 500 mg 4 times daily for 5 days, which she stated she took accordingly. She had vaginal bleeding for 1 day. On the 10th day after surgery, she began experiencing headache, nausea, and vomiting. Her condition progressed to mental-status changes over the next 12 h. She was taken to the emergency room by family members. Her history included recurrent sinusitis and pseudotumor cerebri, both of which were in remission.

The patient was poorly responsive to questions. Her blood pressure was 142/70, pulse 96, respiratory rate 20, and temperature 38.6°C. Her head examination was unremarkable. Nuchal rigidity was present. The heart rhythm was regular with a II/VI systolic ejection murmur. The lungs were clear to auscultation and the abdominal examination was normal. A pelvic examination showed a 4–6-week-size nontender, mobile uterus with a long, closed cervix. No discharge was noted. A neurologic examination revealed generalized hyporeflexia, downgoing Babinski reflex, and a positive Kernig's sign and gag reflex.

The patient's serum hemoglobin was 10.7 g/dl and WBC count was 19,700/mm$^3$. Coagulation studies were normal and the VDRL was nonreactive. Urine, blood, and stool cultures were negative. Computerized tomography revealed no intracranial bleeding or cerebral edema. Sinus and chest X-rays were negative for acute disease. A lumbar puncture was performed and grossly purulent fluid was obtained which yielded the following results: WBC count 10,300/mm$^3$ with 99% neutrophils, glucose 17 g/dl, and protein 420 g/dl. Antigen tests for 10 common organisms were negative except for a positive result for GBS (Directigen, Bectin Dickinson). A spinal fluid culture showed no growth. A cervical culture for GBS done on the 4th hospital day was negative. A cervical swab for *C. trachomatis* was negative. An echocardiogram showed no evidence of valvular disease.

The patient experienced a seizure in the emergency room. An electroencephalogram showed no gross deficits. She was started on dilantin and aqueous penicillin G, 2 million units q 2 h. The patient recovered uneventfully after 14 days of antibiotics. Posttreatment cultures for *N. gonorrhoeae* and GBS were negative.

### DISCUSSION

Early studies have shown the carrier frequency during pregnancy of GBS to be 10–18%.[7] Baker[8] and Dillon et al.[4] reported rates as high as 35%, which varied from one trimester to another. Dillon et al.[4] also demonstrated a higher anorectal than vaginal carriage rate and believed that the rectum was the reservoir for GBS. Subsequently, GBS colonizes the perineum, vagina, and occasionally the urinary tract. These bacteria are sensitive to the penicillins, aminoglycosides, and cephalosporins, but are resistant to tetracycline and its derivatives, which was prescribed for both patients in this report. GBS infections tend to be more prevalent in animals than in humans. The strains of *Streptococcus agalactiae* in animals are biochemically different and considerably more susceptible to tetracycline than human strains are.[9,10]

Most GBS infections in adults occur in postpartum women. GBS is a common cause of early-onset postpartum endometritis and accounts for 10–20% of blood culture isolates of women on an obstetrical service. In addition, GBS is a common cause of bacteriuria during pregnancy and, less commonly, pyelonephritis.[11] Infections in nonpregnant adults include sepsis, pneumonia, skin, soft tissue, and joints as well as endocarditis and meningitis. Infections in nonpregnant adults are commonly associated with underlying conditions such as diabetes, cancer, liver or renal disease, and immunosuppression.

Endometritis is a common complication of first-trimester (0.1–5.2%) and second-trimester (1.5–18%) abortion.[12,13] The most commonly isolated organisms are GBS, *Escherichia coli, N. gonorrhoeae, C. trachomatis,* and *Bacteroides* species. Complications from abortions, including sepsis and death, involving GBS are rare. Of 36 women who died secondary to septic abortions between 1975 and 1977 (legal, illegal, or spontaneous) in the United States, 23 underwent postmortem examinations and only 1 was infected with GBS. Meningitis was not mentioned as a finding in any of the 36 fatal cases.[14] GBS endocarditis following abortion or term delivery, oftentimes fatal, has been reported in more than 20 women.[15,16] Many cases occurred in the preantibiotic era or in patients with preexisting valvular disease.

Only 33 cases of GBS meningitis have been described in adults. In nonpregnant patients, men and women are affected equally and often the meningitis is associated with the underlying medical conditions mentioned previously. Approximately one-third are associated with pregnancy. Two women were reported to have GBS meningitis following term delivery.[10,17] Both women developed symptoms within 48 h of delivery and recovered without sequelae. Of interest, 1 male newborn also developed GBS meningitis. The clinical features of the cases presented here are similar to those previously reported except for the extended time between dilatation and curettage and the development of symptoms in case 2 (10 days). The physical and laboratory findings were consistent with bacterial meningitis. Although CSF cultures were negative in both patients, antigen tests of the CSF supported the diagnosis of GBS meningitis. Directigen is a latex agglutination test used for making the presumptive diagnosis of meningitis caused by a variety of pathogens including *S. agalactiae.* In a study of body fluid specimens of 94 sick infants, including 15 with GBS meningitis, Rench et al.[18] reported a sensitivity of 94.7% and a specificity of 100%. There were no false-positive latex agglutination tests on CSF specimens.

### CONCLUSIONS

As discussed above, life-threatening GBS infections are quite rare. When these infections are treated appropriately, their outcomes are usually favorable. Due to the rarity of GBS infections in nonpregnant women and women undergoing abortion, routine preoperative testing is not indicated.

### REFERENCES

1. Anon: Induced termination of pregnancy before and after Roe v. Wade: Trends in mortality and morbidity of women. JAMA 268:3231–3239, 1992.
2. Hodgson JE, Major B, Portmann K, Quattlebaum FW: Prophylactic use of tetracycline for first trimester abortions. Obstet Gynecol 45:574–578, 1975.
3. McNeeley SG: Pelvic inflammatory disease. Curr Opin Obstet Gynecol 4:682–686, 1992.

4. Dillon HC, Gray E, Pass MA, Gray BM: Anorectal and vaginal carriage of group B streptococci during pregnancy. J Infect Dis 145:794–799, 1982.
5. Franciosi RA, Knotsman JD, Zimmerman RA: Group B streptococcal neonatal and infant infections. J Pediatr 82: 707–718, 1973.
6. Ablow RC, Driscoll SG, Effman EL, Gross I, Jolles CJ, Uauy R, Warshaw JB: A comparison of early onset group B streptococcal neonatal infection and the respiratory distress syndrome of the newborn. N Engl J Med 294:65, 1976.
7. Badri MS, Zawaneh S, Cruz AC, Mantilla G, Baer H, Spellacy WN, Ayoub EM: Rectal colonization with group B streptococcus: Relation to vaginal colonization of pregnant women. J Infect Dis 135:308–312, 1977.
8. Baker CJ: Summary of the workshop on perinatal infections due to group B streptococcus. J Infect Dis 136:137–152, 1977.
9. Bayer AS, Chow AW, Anthony BF, Guze LB: Serious infections in adults due to group B streptococci: Clinical and serotypic characterization. Am J Med 61:498–503, 1976.
10. Lerner PI, Gopalakrishna KV, Wolinsky E, McHenry MC, Tan JS, Rosenthal M: Group B streptococcus (*S. agalactiae*) bacteremia in adults: Analysis of 32 cases and review of the literature. Medicine 56:457–473, 1977.
11. Faro S: Group B beta-hemolytic streptococci and puerperal infections. Am J Obstet Gynecol 139:686–689, 1981.
12. Burkman RT, Atienza MF, King TM: Culture and treatment results in endometritis following elective abortion. Am J Obstet Gynecol 128:556–559, 1977.
13. Hodgson JE, Portmann KC: Complications of 10,453 consecutive first-trimester abortions: A prospective study. Am J Obstet Gynecol 120:802–807, 1974.
14. Grimes DA, Cates W, Selik RM: Fatal septic abortion in the United States, 1975–1977. Obstet Gynecol 57:739–744, 1981.
15. Sexton DJ, Rockson SG, Hempling RE, Cathey CW: Pregnancy-associated group B streptococcal endocarditis: A report of two fatal cases. Obstet Gynecol 66:44S–46S, 1985.
16. Jemsek JG, Gentry LO, Greenberg SB: Malignant group B streptococcal endocarditis associated with saline-induced abortion. Chest 76:695–697, 1979.
17. Grossman J, Tompkins RL: Group B beta-hemolytic streptococcal meningitis in mother and infant. N Engl J Med 290:387–388, 1974.
18. Rench MA, Metzger TG, Baker CJ: Detection of Group B streptococcal antigen in body fluids by a latex-coupled monoclonal antibody assay. J Clin Microbiol 20:852–854, 1984.

## References

[PDF_00273] Ablow R. C., Driscoll S. G., Effmann E. L., Gross I., Jolles C. J., Uauy R., Warshaw J. B. (1976). A comparison of early-onset group B steptococcal neonatal infection and the respiratory-distress syndrome of the newborn.. N Engl J Med.

[PDF_00278] Badri M. S., Zawaneh S., Cruz A. C., Mantilla G., Baer H., Spellacy W. N., Ayoub E. M. (1977). Rectal colonization with group B streptococcus: relation to vaginal colonization of pregnant women.. J Infect Dis.

[PDF_00285] Bayer A. S., Chow A. W., Anthony B. F., Guze L. B. (1976). Serious infections in adults due to group B streptococci. Clinical and serotypic characterization.. Am J Med.

[PDF_00296] Burkman R. T., Atienza M. F., King T. M. (1977). Culture and treatment results in endometritis following elective abortion.. Am J Obstet Gynecol.

[PDF_00267] Dillon H. C., Gray E., Pass M. A., Gray B. M. (1982). Anorectal and vaginal carriage of group B streptococci during pregnancy.. J Infect Dis.

[PDF_00293] Faro S. (1981). Group B beta-hemolytic streptococci and puerperal infections.. Am J Obstet Gynecol.

[PDF_00270] Franciosi R. A., Knostman J. D., Zimmerman R. A. (1973). Group B streptococcal neonatal and infant infections.. J Pediatr.

[PDF_00302] Grimes D. A., Cates W., Selik R. M. (1981). Fatal septic abortion in the United States, 1975-1977.. Obstet Gynecol.

[PDF_00312] Grossman J., Tompkins R. L. (1974). Group B beta-hemolytic streptococcal meningitis in mother and infant.. N Engl J Med.

[PDF_00260] Hodgson J. E., Major B., Portmann K., Quattlebaum F. W. (1975). Prophylactic use of tetracycline for first trimester abortions.. Obstet Gynecol.

[PDF_00299] Hodgson J. E., Portmann K. C. (1974). Complications of 10,453 consecutive first-trimester abortions: a prospective study.. Am J Obstet Gynecol.

[PDF_00309] Jemsek J. G., Gentry L. O., Greenberg S. B. (1979). Malignant group B streptococcal endocarditis associated with saline-induced abortion.. Chest.

[PDF_00289] Lerner P. I., Gopalakrishna K. V., Wolinsky E., McHenry M. C., Tan J. S., Rosenthal M. (1977). Group B streptococcus (S. agalactiae) bacteremia in adults: analysis of 32 cases and review of the literature.. Medicine (Baltimore).

[PDF_00263] McNeeley S. G. (1992). Pelvic inflammatory disease.. Curr Opin Obstet Gynecol.

[PDF_00315] Rench M. A., Metzger T. G., Baker C. J. (1984). Detection of group B streptococcal antigen in body fluids by a latex-coupled monoclonal antibody assay.. J Clin Microbiol.

[PDF_00305] Sexton D. J., Rockson S. G., Hempling R. E., Cathey C. W. (1985). Pregnancy-associated group B streptococcal endocarditis: a report of two fatal cases.. Obstet Gynecol.

[PDF_00282] (1977). Summary of the workshop on perinatal infections due to group B Streptococcus.. J Infect Dis.

